# Programming Nanostructure Formation Through Furin‐Triggered Isopeptide Conversion and Peptide Self‐Assembly

**DOI:** 10.1002/mabi.202500427

**Published:** 2025-10-25

**Authors:** Sarah Chagri, Jana Fetzer, Patrick Roth, Albin Lahu, Nico Alleva, Jian Zhang, Manfred Wagner, Shutian Si, Ingo Lieberwirth, Katharina Landfester, David Y. W. Ng, Tanja Weil

**Affiliations:** ^1^ Department For the Synthesis of Macromolecules Max Planck Institute For Polymer Research Mainz Germany; ^2^ Department For the Physical Chemistry of Polymers Max Planck Institute For Polymer Research Mainz Germany

**Keywords:** enzyme‐responsive peptides, furin, nanostructures, self‐assembly

## Abstract

The controlled bioresponsive formation of synthetic nanostructures requires the precise chemical design of precursor molecules that undergo stimulus‐induced chemical conversion and subsequent self‐assembly. In this study, we present an enzymeresponsive kinked isopeptide containing the recognition sequence RVRR of the protease furin, an enzyme that is overexpressed in many cancers. Despite the unnatural kinked structure of the isopeptide, we show that it is enzymatically cleaved, causing its rearrangement into a linear peptide capable of forming fibrillar nanostructures. We investigate the kinetics of this enzymatic transformation and compare it to a non‐cleavable control isopeptide bearing a scrambled RRRV sequence. The material properties of the linear fiber‐forming peptide are characterized using circular dichroism spectroscopy, fluorescence, and electron microscopy, as well as nuclear magnetic resonance spectroscopy. This study provides insights into the furin‐induced transformation of a kinked isopeptide for the controlled formation of nanostructures and highlights the potential of enzyme‐triggered isopeptide systems for the design of functional materials.

## Graphical abstract

A kinked *iso*peptide with a furin recognition sequence (RVRR) is designed to respond to the protease furin by transforming into a linear peptide that self‐assembles into elongated nanofibers. This study shows how enzyme‐triggered transformation of a synthetic kinked *iso*peptide enables precise and controlled nanostructure formation, offering new strategies for advanced functional material design and biological applications.

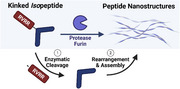

## 1 Introduction

Over the past decades, bioresponsive material transformation has emerged as a powerful strategy for the controlled formation of synthetic nanostructures under biologically relevant conditions [1, 2, 3, 4]. These transformations rely on endogenous chemical or enzymatic triggers to induce molecular changes that lead to supramolecular assembly formation. The ability to control such stimuli‐responsive processes opens promising opportunities for applications ranging from intracellular nanomaterials to tissue engineering. In particular, the precise control over structure formation through stimulus‐induced chemical conversion is of increasing interest from a bottom‐up synthetic biology perspective, enabling the design of systems that mimic or interface with complex biological organisms [5, 6].

Three key factors typically govern the efficiency and outcome of bioresponsive structure formation: (1) the abundance and specificity of the biological trigger, (2) the kinetics of the conversion process from precursor to active building block, and (3) the supramolecular characteristics of the resulting assemblies. The nature and local concentration of the trigger are critical, as a wide range of stimuli, such as redox conditions, pH value changes, or enzyme activity, have all been used for material transformations, often leading to vastly different structural and functional outcomes [1]. Typically, chemical triggers that are abundant throughout the cell, such as the ubiquitous reducing agent glutathione [7], cause immediate transformation of the assembly precursor upon cell entry, resulting in a high local concentration of the self‐assembling monomer [8, 9].

Using naturally occurring enzymes to trigger bioresponsive transformation offers the advantage of achieving a controlled conversion over time that is dependent on local enzyme activity [10, 11].

**FIGURE 1:**
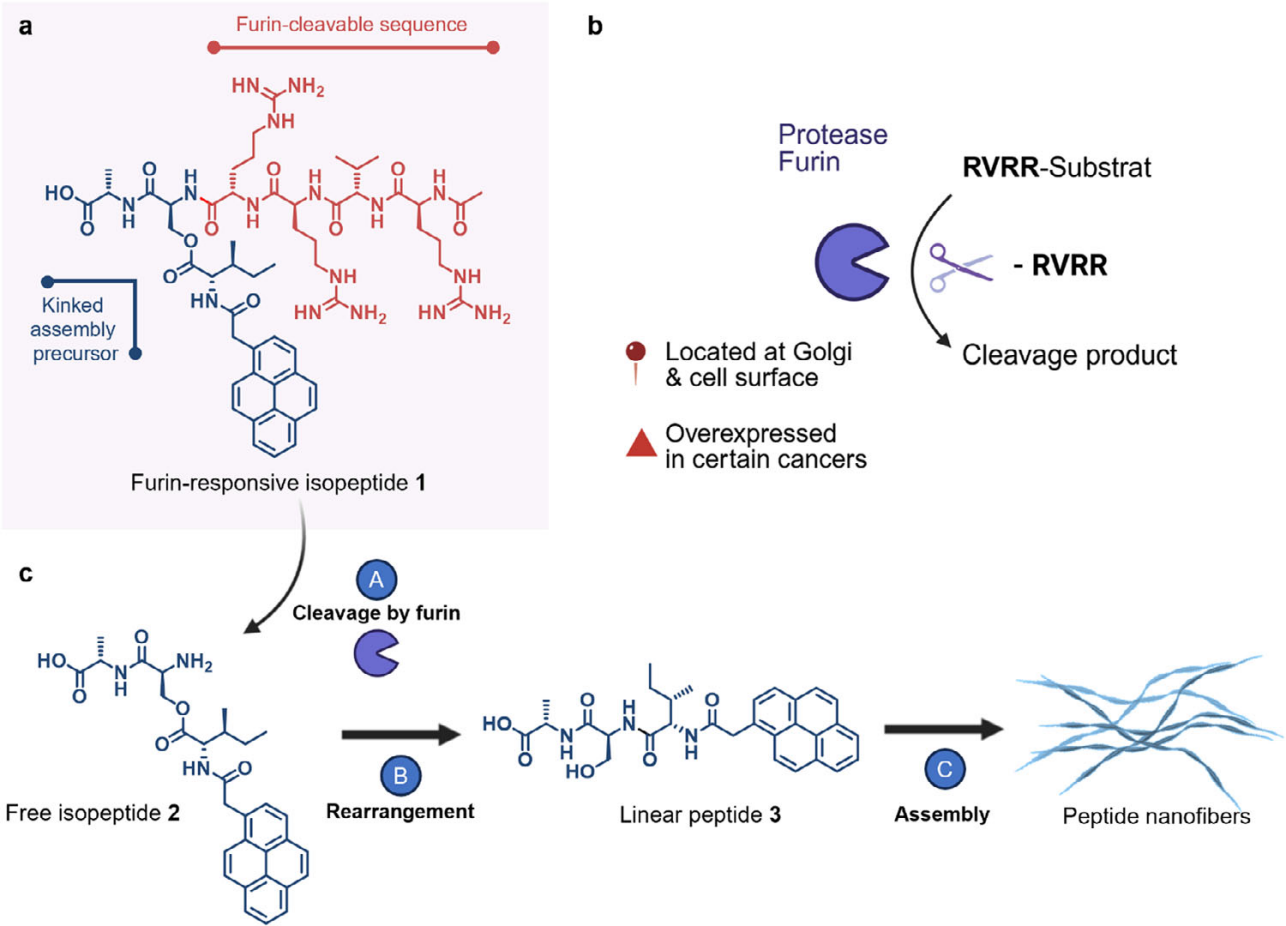
Chemical structure of furin‐responsive isopeptide **1**, subcellular localization of furin, and enzyme‐induced multistep reaction cascade. (a) The furin‐responsive kinked isopeptide **1** is composed of a pro‐assembling iso‐tripeptide with pyrene as a fluorescent *N*‐terminal substituent and the enzyme‐cleavable sequence RVRR. (b) Furin is a Golgi‐associated endoprotease capable of cleaving downstream of its recognition sequence RVRR. (c) The isopeptide assembly precursor **1** can be cleaved by furin (A), resulting in the rearrangement of the free isopeptide **2** (B). The linear peptide **3** can assemble into fibrillar nanostructures (C).

One example of such an enzyme is the protease furin, which is typically located at the Golgi apparatus in eukaryotic cells and on cell surfaces. Furin cleaves substrates downstream of the polybasic recognition sequence RX(R/K)R (Figure 1b) [12] and is overexpressed in many cancer cell lines [13], making it an interesting target for triggering bioresponsive material transformation [14, 15, 16, 17].

Beyond the identity of the trigger, the mechanism and complexity of the transformation process itself significantly influence structural dynamics. While many systems rely on a single‐step cleavage to release the assembling monomer, multi‐step conversion pathways offer more kinetic control, allowing each step to be tailored to a specific environment or biological cure [18].

Herein, we introduce a furin‐responsive kinked isopeptide designed for the controlled formation of nanostructures via the enzyme‐triggered multistep transformation into a self‐assembling linear peptide, which offers a new level of complexity due to an additional rearrangement step before forming the self‐assembling monomer (Figure 1a,c). This additional step not only represents a different mechanism of monomer generation but also allows more options for the transformation kinetics to be modulated by the chemical environment, potentially offering finer control over assembly timing and localization. The enzymatic cleavage and subsequent conversion of the isopeptide were monitored using time‐dependent high‐performance liquid chromatography (HPLC) and occurred within a few hours of mixing with enzyme‐containing buffered solution. To characterize the supramolecular and structural properties of the resulting linear peptide monomer, we employed a range of techniques, including fluorescence microscopy, transmission electron microscopy (TEM), cryogenic transmission electron microscopy (cryo‐TEM), circular dichroism (CD), and nuclear magnetic resonance (NMR) spectroscopy. These allowed us to identify defined nanofibrillar architectures of the linear peptide resulting from the enzymatic transformation of the isopeptides. Our findings serve as a proof‐of‐concept for the use of enzyme‐responsive kinked isopeptide‐based systems for the controlled formation of peptide nanostructures.

## 2 Results and Discussion

### 2.1 Design, Synthesis, and Transformation of Isopeptides

**FIGURE 2:**
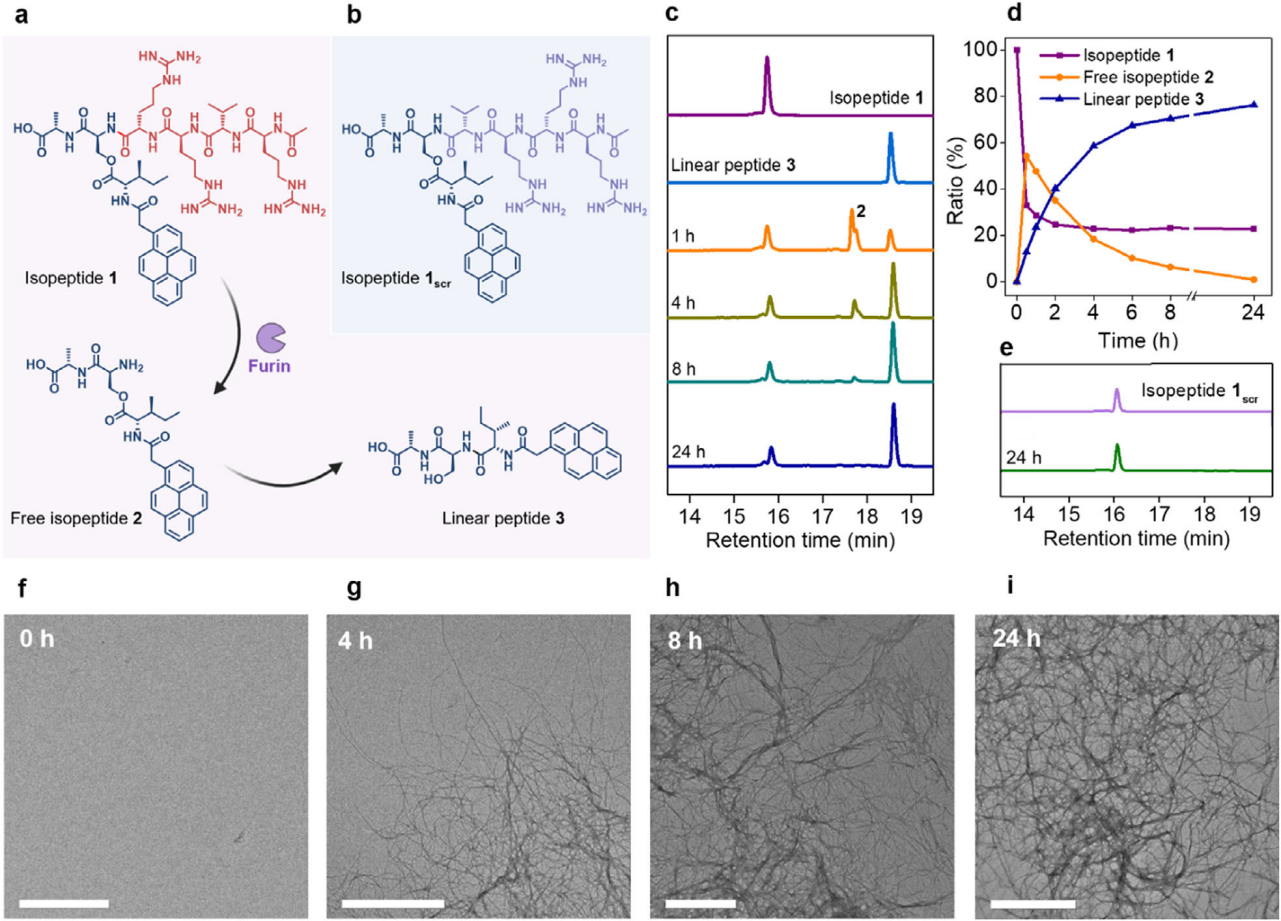
Analysis of furin‐induced conversion of an enzyme‐responsive isopeptide. (a) Reaction scheme of furin‐induced cleavage of isopeptide **1** downstream of the RVRR cleavage site, resulting in free isopeptide **2,** which subsequently undergoes a rearrangement via *O,N* acyl shift to form linear peptide **3**. (b) Chemical structure of non‐cleavable control isopeptide **1_scr_** (RRRV). (c) HPLC study of the kinetics of enzyme‐induced conversion of isopeptide **1** (100 µm) in the presence of the protease furin (1 nmol peptide/U) in buffered solution over time (HEPES (100 mm), CaCl_2_ (1 mm), TCEP (1 mm)). (d) Molar ratio of isopeptide **1**, free isopeptide **2,** and linear peptide **3** over time based on the peak intensity at 340 nm. (e) HPLC study of the scrambled control isopeptide **1_scr_** in the presence of furin in a buffered solution. Due to the scrambled enzyme‐recognition sequence (RRRV), no enzymatic cleavage was detected. (f–i) Transmission electron micrographs of reaction solution of isopeptide **1** (200 µm) in the presence of furin in buffered solution (HEPES (100 mm), CaCl_2_ (1 mm), TCEP (1 mm)) after incubation for 0 h (f), 4 h (g), 8 h (h) and 24 h (i) at 37°C. Scale bars: 500 nm.

The furin‐responsive isopeptide **1** consists of three main structural components: (1) the furin‐cleavable sequence RVRR, (2) a pro‐assembling iso‐tripeptide, and (3) the pyrene moiety that is necessary for self‐assembly and fluorescence (Figure 2a). Additionally, we prepared the non‐cleavable isopeptide **1_scr_** with a scrambled enzyme recognition sequence (RRRV) as a control compound (Figure 2b). The synthesis of these isopeptides was conducted via solid‐phase‐supported peptide synthesis (Scheme S1).

To investigate the furin‐induced transformation of isopeptide **1**, we analyzed the kinetics of this multistep reaction cascade in the presence of commercial furin (1 nmol peptide/U) using HPLC (Figure 2c and Figure S10). Isopeptide **1** was dissolved at a concentration of 100 µm in a buffer solution optimized for the enzyme furin (HEPES (100 mm), CaCl_2_ (1 mm), TCEP (1 mm)) and incubated at 37°C. By analyzing the mixture at various time points, we observed a rapid degradation of isopeptide **1** (*t*_R_ = 15.74 min), with the signal for the linearized peptide **3** (*t*_R_ = 18.54 min) already appearing within the first hour. A peak with a retention time of 17.65 min was attributed to the intermediate free isopeptide **2**, which had undergone enzymatic cleavage but had not yet rearranged into linear peptide **3**. By 24 h, 76% of isopeptide **1** had transformed into the final product, the linear peptide **3**. The observation of less than 100% conversion could be attributed to the accumulation of formed fibers, which might sequester free isopeptide monomers, rendering the cleavage motifs inaccessible. Analysis of the reaction solution at different time points via transmission electron microscopy (TEM) revealed the formation of fibrillar nanostructures already after 4 h of incubation (Figure 2g and Figure S11). After 8 and 24 h of incubation, when the majority of isopeptide **1** is converted to the linear peptide **3** according to the HPLC kinetics analysis, bundled networks of peptide fibers were observed in the TEM analysis (Figures 2h,i and Figure S11). In contrast to the kinetics analysis of the degradation of isopeptide **1**, the control isopeptide **1_scr_**, which contains a scrambled version of the furin‐recognition sequence (RRRV instead of RVRR), showed no furin‐induced degradation over the 24‐h period (*t*_R_ = 16.07 min) (Figure 2e). Overall, these results demonstrate that furin effectively cleaves downstream of the RVRR sequence even within the branched isopeptide **1**, despite the presence of an ester bond on the serine side chain connected to a bulky pyrene‐modified isoleucine. This experiment provides proof of concept for the enzyme‐triggered transformation of a kinked isopeptide into a linearized peptide, which can function as a supramolecular monomer for programmed structure formation. The combination of the kinetics analysis of the enzyme‐induced conversion of isopeptide **1** into linear peptide **3** with the visualization of peptide structures formed by **3** via TEM supports the idea that the furin‐induced conversion of a kinked assembly precursor can lead to in situ structure formation.

### 2.2 Analysis of Peptide Nanostructures

Next, the supramolecular behavior and structure formation of linear peptide **3** were studied using CD and NMR spectroscopy, as well as fluorescence and electron microscopy.

**FIGURE 3:**
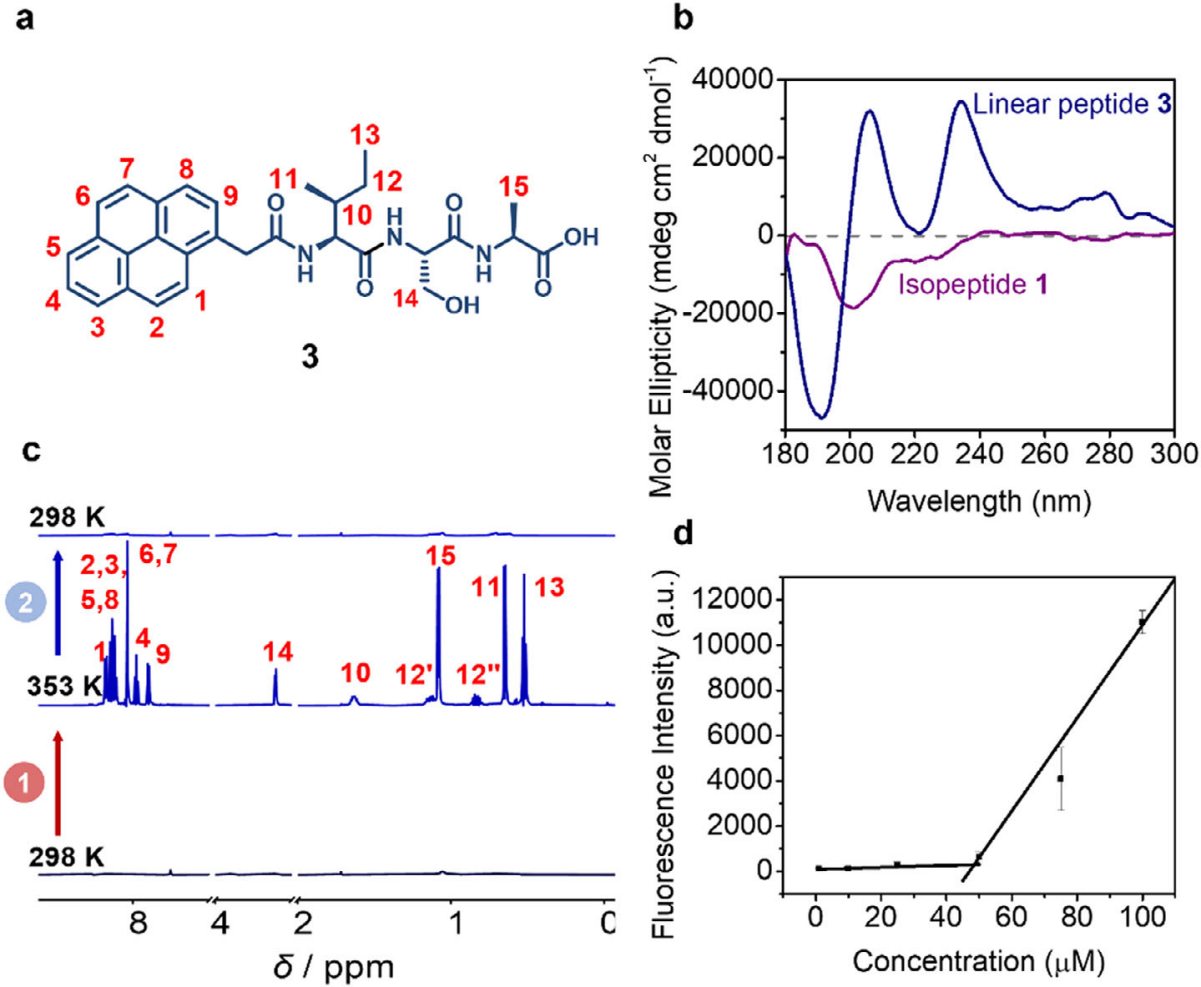
Supramolecular characterization of linear peptide **3**. (a) Chemical structure of linear peptide **3** with labeled protons. (b) Circular dichroism (CD) spectra of linear peptide **3** and kinked isopeptide **1** (100 µm) in phosphate buffer (10 mm, pH 7.4). (c) ^1^H‐NMR analysis of the self‐assembly behavior of linear peptide **3** (1 mg/mL) in deuterated phosphate buffer (50 mm) and DMSO‐*d*_6_ (9:1) at 298 K (bottom), after heating to 353 K (middle), and after cooling back to 298 K (top). (d) Proteostat aggregation assay of linear peptide **3** in DPBS and DMSO (9:1). The critical aggregation concentration of linear peptide **3** was determined to be 48 µm by calculating the intersection of the linear fits.

Temperature‐dependent ^1^H‐NMR spectroscopy provided insights into the dynamic assembly of the linear peptide **3**. The spectrum measured at room temperature (298 K) revealed significant peak broadening, which is characteristic of supramolecular interactions between peptide monomers in aqueous solution (deuterated phosphate buffer (50 mm) and DMSO‐*d*_6_ (9:1)) (Figures 3a,c and S12). Upon heating to 353 K, the appearance of sharp signals corresponding to the aromatic pyrene moiety and side chain groups suggested an increased amount of monomeric peptide **3** in solution at elevated temperatures.

CD spectroscopy gives information on the secondary structure and chirality of peptides. While the spectrum of the kinked isopeptide **1** displayed a single minimum at 201 nm, indicative of an overall random coil structure, the CD spectrum of linear peptide **3** exhibited a more complex profile with a minimum at 191 nm and two maxima at 206 and 233 nm (Figure 3b). The negative band at 191 nm can be attributed to the π→π^*^ transition of peptide bonds within the backbone, while the strong positive band at 206 nm likely reflects π→π^*^ transitions modulated by the *N*‐terminal pyrene moiety. The positive signal at 233 nm is suggestive of the n→π^*^ electronic transition associated with the aromatic pyrene group, underscoring its significant role in the overall conformation of the peptide. To determine the critical aggregation concentration of linear peptide **3**, a fluorescence‐based Proteostat aggregation assay was conducted. Solutions of the linear peptide **3** at varying concentrations were prepared in DPBS and DMSO (9:1) and incubated for over 24 h, before adding the aggregation‐sensitive Proteostat dye. Fluorescence measurements revealed a critical aggregation concentration of approximately 48 µm for the linear peptide **3** (Figure 3d). To ensure that isopeptide **1** does not exhibit aggregation at concentrations up to 250 µm, a Proteostat assay was performed for this compound, too. The results showed that, unlike linear peptide **3**, isopeptide **1** did not display similar aggregation behavior and instead produced a low signal comparable to the solvent‐only control (Figure S14).

**FIGURE 4:**
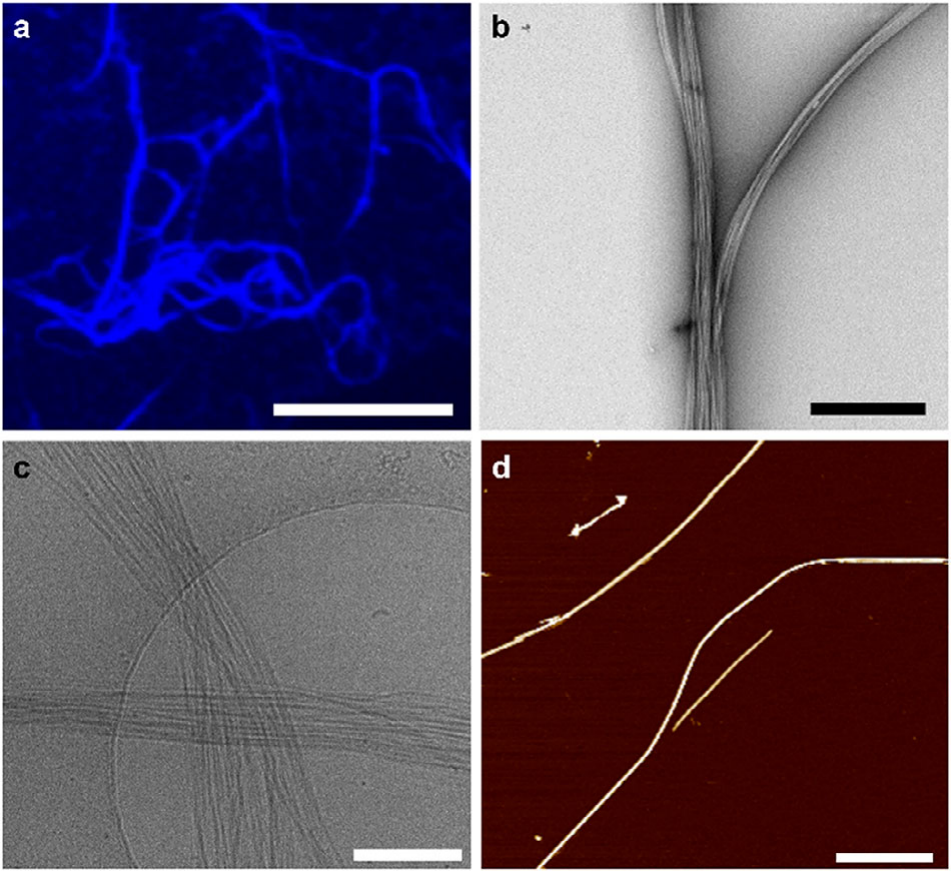
Characterization of nanofiber formation by linear peptide **3**. (a) Fluorescence microscopy image of linear peptide **3** (100 µm) in DPBS and DMSO (9:1). Scale bar: 50 µm. (b) Dry‐state TEM image showing nanofiber bundles formed by linear peptide **3** (100 µm) in DPBS (pH 7.4) and DMSO (9:1). Scale bar: 500 nm. (c) Cryo‐EM image of peptide nanofibers formed by linear peptide **3** (500 µm) in DPBS (pH 7.4) and DMSO (9:1). Scale bar: 250 nm. (d) AFM image of peptide nanofibers formed by linear peptide 3 (250 µm) in DPBS (pH 7.4) and DMSO (9:1). Scale bar: 270 nm.

Fluorescence microscopy of an aqueous solution of linear peptide **3** at 100 µm in phosphate buffer (pH 7.4) and DMSO (9:1) showed the spontaneous formation of thin fibers extending several micrometers in length (Figure 4a). Cryo‐electron microscopy (cryo‐EM) images confirmed the presence of elongated fibrillar nanostructures at both 500 and 100 µm peptide concentrations (Figure 4c). Transmission electron microscopy (TEM) in the dry state also visualized bundled fibrillar assemblies (Figure 4b and Figure S13). Atomic force microscopy (AFM) was used to further characterize the nanofibers, revealing elongated fibers that extend over several hundred nanometers in length. (Figure 4d and Figure S15). The observed formation of peptide nanostructures via the assembly of linear peptide **3** is a critical prerequisite for their application in enzyme‐induced peptide nanostructure formation within biological contexts.

## 3 Conclusions

This study demonstrates the successful design, synthesis, and characterization of a furin‐responsive kinked isopeptide that undergoes enzyme‐triggered transformation into a linear self‐assembling peptide. We show that furin efficiently catalyzes the conversion of the isopeptide into a linear peptide despite the kinked structure of the precursor molecule. The linear peptide resulting from the enzyme‐induced transformation can assemble into elongated fibrillary‐nanostructures with a distinct nanoscale morphology, which was studied using various microcopy characterization techniques. Leveraging proteolytic enzyme activity for the conversion of a kinked isopeptide to achieve controlled nanostructure formation offers significant potential for biological applications of self‐assembling peptides, such as immunotherapy [5], controlled drug delivery [6], and tissue engineering [19]. Therefore, future exploration should focus on treating furin‐overexpressing cells with the furin‐responsive isopeptides to analyze the conversion and structure formation, as well as the impact on cellular function. Beyond the chemical design presented here, incorporating other functionalities and varying the peptide sequence could enable the generation of new nanomaterials with diverse fluorescent properties, different supramolecular characteristics, and morphologies. Overall, enzyme‐responsive isopeptides could offer a range of opportunities for controlled in situ structure formation in biological environments.

## 4 Experimental Section

See Supporting Information

## Author Contributions

S.C., D.Y.W.N., and T.W. conceived the research concept. S.C. and J.F. conducted the synthesis and characterization of peptides and associated molecules, as well as the HPLC study and Proteostat assay. S.C. performed the fluorescence microscopy measurements. J.F. conducted the CD measurements. P.R. and A.L. performed the TEM measurements. S.S., I.L., and K.L. performed cryo‐EM measurements and interpreted the results. N.A. and J.Z. performed AFM measurements. M.W. performed NMR analysis. D.Y.W.N. and T.W. supervised the project. All authors have read and approved the final manuscript. All the authors have read and commented on the manuscript.

## Conflicts of Interest

The authors declare no conflicts of interest.

## Supporting information




**Supporting File**: mabi70095‐sup‐0001‐SuppMat.docx.

## Data Availability

The data that support the findings of this study are available from the corresponding authors upon reasonable request.
