# Supplementary material for: Programming Nanostructure Formation Through Furin‐Triggered Isopeptide Conversion and Peptide Self‐Assembly
Source: Macromol Biosci. 2025 Oct 25;26(1):e00427. doi: 10.1002/mabi.202500427 (PMC12829517; doi:10.1002/mabi.202500427)
Supplement: Supplementary file 1 — Supporting File: mabi70095‐sup‐0001‐SuppMat.docx. [file MABI-26-e00427-s001.docx]

Supporting Information

**Programming Nanostructure Formation through Furin-Triggered Isopeptide Conversion and Peptide Self-Assembly**

*Sarah Chagri, ‡ Jana Fetzer, ‡ Patrick Roth, Albin Lahu, Nico Alleva, Jian Zhang, Manfred Wagner, Shutian Si, Ingo Lieberwirth, Katharina Landfester, David Y. W. Ng* and Tanja Weil**

**Table of Content**

1. General Information 18

1.1. Materials 18

1.2. Instruments 18

1.2.1. Microwave Peptide Synthesizer 18

1.2.2. High-Performance Liquid Chromatography (HPLC) 18

1.2.3. Liquid Chromatography-Mass Spectrometry (LC-MS) 18

1.2.4. Matrix-Assisted Laser Desorption/Ionisation-Time of Flight Mass Spectrometry (MALDI-TOF) 18

1.2.5. Nuclear Magnetic Resonance Spectroscopy (NMR) 19

1.2.6. Circular Dichroism Spectroscopy (CD) 19

1.2.7. Transmission Electron Microscopy (TEM) 19

1.2.8. Cryogenic Transmission Electron Microscopy (cryo-TEM) 19

1.2.9. Fluorescence Microscopy 19

1.2.10. Atomic Force Microscopy (AFM) 19

2. Synthesis 20

2.1. Synthesis of Isopeptides 20

2.1.1. Synthesis of Isopeptides with RVRR or RRRV Sequence 20

2.1.2. Solid Phase Supported Synthesis of Isopeptides 23

2.2 Synthesis of Linear Py-ISA 3 25

3. Furin-Induced Transformation of Isopeptides 28

3.1. General procedure of HPLC Kinetic Analysis 28

3.2. General Procedure of TEM Kinetic Analysis 29

4. Analysis of Secondary Structure and Self-Assembly Behavior 30

4.1. Temperature-dependent ^1^H-NMR Analysis of Linear Py-ISA 3 30

4.2. CD Spectra of Py-ISA 3 and Kinked Assembly Precursor 1 30

4.3. TEM Analysis of Linear Py-ISA 3 and Kinked Assembly Precursor 1 31

4.4. Cryo-TEM Analysis of Linear peptide 3 31

4.5. Proteostat Aggregation Assay 31

4.6. AFM 32

**1. General Information**

**1.1. Materials**

Reagents and solvents were purchased from commercial sources and were used without further purification. Peptide synthesis grade reagents were used for synthesizing the peptides. HPLC was performed using acetonitrile (CH_3_CN) in HPLC grade (containing 0.1% trifluoroacetic acid (TFA)) and water for HPLC (containing 0.1% TFA) and reactions was obtained from a Millipore purification system. Flash column chromatography was carried out using Macherey-Nagel silica gel 0.04–0.063 mm. Furin (2,000 units/mL) was purchased from *New England Biolabs*.

**1.2. Instruments**

**1.2.1. Microwave Peptide Synthesizer**

Linear peptides were synthesized in a Liberty Blue Automated Microwave Peptide Synthesizer by CEM Corporation.

**1.2.2. High-Performance Liquid Chromatography (HPLC)**

The peptides were purified by preparative HPLC using a setup by Shimadzu. For purification either a Zorbax Eclipse XDB-C18 HPLC column (9.4 × 250 mm, 5 μm) was used at a flowrate of 4 ml/min or a Phenomenex Gemini 5 μm NX-C18 110 Å 150 × 30 mm was used at a flowrate of 25 ml/min. For analytical measurements a Waters Atlantis T3, C18 column (5 μm, 100 Å, 4.6 x 100 mm) was used.

**1.2.3. Liquid Chromatography-Mass Spectrometry (LC-MS)**

Compounds were analyzed by HPLC-ESI-MS on a LC-MS 2020 by Shimadzu using a Kinetex 2.6 μm EVO C18 100 Å LC 50 × 2.1 mm column. MilliQ water acidified with 0.1% formic acid and CH_3_CN were used as solvents for all measurements. The solvent gradient started with 5% CH_3_CN and 95% water. This solvent ratio was kept constant for 2 min, then the CH_3_CN content was linearly increased to 95% in 14 min. Data were processed in *LabSolutions* and *OriginPro 9*.

**1.2.4. Matrix-Assisted Laser Desorption/Ionisation-Time of Flight Mass Spectrometry (MALDI-TOF)**

All MALDI-TOF spectra were recorded on either a rapifleX MALDI-TOF/TOF from Bruker or MALDI Synapt G2-SI from Waters. Samples were mixed with a saturated solution of the matrix S7 α-cyano-4-hydroxycinnamic acid (CHCA) in H_2_O/CH_3_CN 1/1 + 0.1% TFA. Data processing was performed in *mMass* and *OriginPro 9*.

**1.2.5. Nuclear Magnetic Resonance Spectroscopy (NMR)**

NMR spectra of small molecules and peptides were recorded on a Bruker Avance 400 MHz spectrometer and an Avance III 850 MHz spectrometer. The solvent signal was used as a reference (deuterated chloroform CDCl_3_ δ =7.26 ppm for ^1^H, 77.16 ppm for ^13^C, D_2_O 4.65 ppm and for DMSO-d_6_ 2.50 ppm and 39.52 ppm respectively). The data was processed in *MestReNova*.

**1.2.6. Circular Dichroism Spectroscopy (CD)**

CD spectra were recorded on a JASCO J-1500 spectrometer in a 0.1 cm High Precision Cell by *HellmaAnalytics*. The recorded data was processed in Spectra Analysis by *JASCO* and *OriginPro9*.

**1.2.7. Transmission Electron Microscopy (TEM)**

TEM images of the peptide samples were recorded on a JEOL 1400 transmission electron microscope at a voltage of 120 kV. Formvar/carbon-film coated copper grids (300 mesh) by Plano GmbH were used to prepare the samples. The images were processed in *Fiji ImageJ*.

**1.2.8. Cryogenic Transmission Electron Microscopy (cryo-TEM)**

For cryo-TEM examination the samples were vitrified using a Vitrobot Mark V (Thermo Fisher, Hilsboro Oregon) plunging device. 3 µl of the sample dispersion was applied to a Quantifoil® R 1.2/1.3 300 Mesh, Cu, that has been glow discharged in an oxygen plasma cleaner (Diener Nano®, Diener electronic, Germany) shortly before. After removing excess sample solution with a filter paper, the grid is immediately plunged into liquid ethane. For the subsequent examination, the specimen is transferred to a TEM (FEI Titan Krios G4) keeping cryogenic conditions. Conventional TEM imaging was done using an acceleration voltage of 300 kV. Micrographs were acquired with a 4k Direct Electron Detection Camera (Gatan K3) under low dose conditions.

**1.2.9. Fluorescence Microscopy**

Fluorescence images of peptide nanostructures were taken using a Keyence BZ-X810 fluorescence microscope using a 20x LD PH lens and a DAPI filter: Ex.: 360/40 nm, Em.: 460/50 nm.

**1.2.10. Atomic Force Microscopy (AFM)**

40 μL of a [250 μM] Py-ISA peptide **3** solution was dropped on a freshly peeled mica. After waiting for 10 min at room temperature to adsorb, additional 260 μL PBS containing 10 % DMSO was added gently. Peptide samples were imaged by using an AFM Instrument (Dimension Fast Scan Bio, Bruker Corporation, Inc.) in liquid tapping mode with Fastscan D tips. The scan rates were between 1 and 3 Hz. Obtained images were further analyzed by using *Nanoscope Analysis 1.9* software.

**2. Synthesis**

**2.1. Synthesis of Isopeptides**

**2.1.1. Synthesis of Isopeptides with RVRR or RRRV Sequence**

**Scheme S1:** a) Synthesis of pyrene-modified isoleucine: i) PyBOP, DIPEA, DCM, 3 h, RT; ii) TFA,DCM (1:1), 4 h, RT. b) Synthesis of isopeptides with RVRR and RRRV sequences: iii) DIC, DMAP, DCM, overnight, RT; iv) TFA, TIPS, H_2_O (95:2.5:2.5)h, RT.

**Synthesis of Pyrene-modified Isoleucine (Py-Ile) 5**

1-Pyreneacetic acid (130.15 mg, 0.50 mmol, 1.0 eq.) was dissolved in dry DMF (5 mL), then DIPEA (0.50 mL, 3.00 mmol, 6.0 eq.) was added. Subsequently, PyBOP (520.4 mg, 1.00 mmol, 2.0 eq.) and ^t^Bu-L-Ile × HCl (111.87 mg, 0.50 mmol, 1.0 eq.) were dissolved in dry DMF (5 mL) and added to solved 1-pyreneacetic acid. The reaction mixture was stirred at room temperature for 3 h before the solvent was removed under reduced pressure. The crude ^t^Bu‑protected product was purified via column chromatography (CH/EA, 2:1) yielding Py-Ile as a colorless solid (191.5 mg, 0.44 mmol, 89%). Last, the *^t^*Bu‑protecting group was removed by adding a mixture of DCM/TFA (10 mL, 1:1). After stirring at room temperature for 3 h the solvent was removed under reduced pressure and the product **5** was used for further synthesis.

**^1^H-NMR** (400 MHz, DMSO-*d*_6_) *δ*/ppm = 8.53 (d, *J* = 8.5 Hz, 1H, H-11), 8.43 (d, *J* = 9.2 Hz, 1H, H-aromat), 8.28 (ddd, *J* = 7.8, 4.8, 1.2 Hz, 2H, H-aromat), 8.26 – 8.17 (m, 2H, H-aromat), 8.15 (s, 2H, H-aromat), 8.10 – 8.01 (m, 2H, H-aromat), 4.35 (d, *J* = 14.7 Hz, 1H, H-10), 4.30 – 4.18 (m, 2H, H-10’, H-12), 1.96 – 1.73 (m, 1H, H-13), 1.55 – 1.40 (m, 1H, H-14), 1.34 – 1.17 (m, 1H, H-14’), 0.88 – 0.83 (m, 6H, H‑15, H‑16).

**^13^C-NMR** (101 MHz, DMSO-d_6_) *δ*/ppm = 173.13, 170.40, 131.20, 130.84, 130.38, 129.70, 128.95, 128.71, 127.42, 127.10, 126.83, 126.20, 125.09, 124.93, 124.72, 124.16, 124.10, 123.94, 56.39, 36.49, 24.74, 15.68, 11.29.

**ESI** m/z: [M+H]^+^ 374.2 (calc. 374.2), [2M+H]^+^ 747.5 (calc. 747.3).


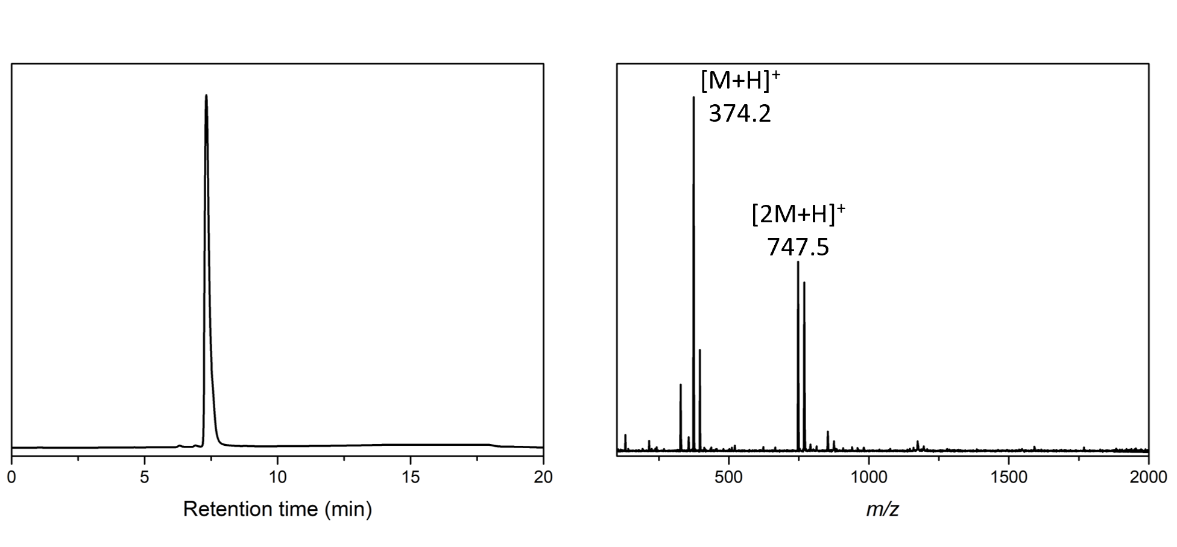


**Figure S1:** LC-MS data of Py-Ile **5**. Left LC trace (254 nm) with *t*_R_= 7.33 min. Right Convoluted ESI-MS spectrum.

**Figure S2:** ^1^H-NMR spectrum (400 MHz, DMSO-*d*_6_, 298 K) of Py-Ile **5**.

**Figure S3:** ^1^H,^1^H-COSY NMR spectrum (400 MHz, DMSO-*d*_6_, 298 K) of Py-Ile **5**.

**Figure S4:** ^13^C-NMR spectrum (101 MHz, DMSO-*d*_6_, 298 K) of Py-Ile **5**.

**2.1.2. Solid Phase Supported Synthesis of Isopeptides**

The peptides were synthesized using a microwave assisted solid-phase peptide synthesizer. The Wang resin preloaded with Fmoc-Ala (0.705 g, 0.5 mmol) was covered and swollen in DMF for 1 h. Before every coupling step, the *N*‑terminal Fmoc group was cleaved by two deprotection steps using 20% piperidine in DMF (3 ml) for 15 s and 50 s at 75 °C and 90 °C, respectively. Fmoc-Ser(OH), Fmoc-Arg(Pbf) (2x), Fmoc-Val and Ac-Arg(Pbf) (5 eq. in 1.25 ml) were coupled for 15 s and 110 s at 75 °C and 90 °C respectively using DIC (0.5 M) and Oxyma (1 M) in a total of 2.75 ml DMF. For Fmoc-Arg(Pbf) and Ac-Arg(Pbf) the coupling steps were performed as double couplings with additional washing steps.The resin was removed from the reaction vessel and washed with DMF and DCM. Before the next coupling step the resin was halved.
Py-Ile (102.70 mg, 0.275 mmol, 1.1 eq.) and DMAP (30.54 mg, 0.25 mmol, 1.0 eq.) were dissolved in dry DCM (5 mL), then DIC (0.16 mL, 1.00 mmol, 4 eq.) was added to the solution. Dry DCM (3 mL) was added to the resin (0.25 mmol) loaded with Ac-RVRRSA and the previously prepared Py-Ile solution was added dropwise. The resin was shaken over night at room temperature. The peptide sequence was cleaved from the resin by adding a mixture of TFA (4.75 mL, 95 %), TIPS (0.125 mL, 2.5 %) and H2O (0.125 mL, 2.5 %) and shaken for 2.5 h at room temperature. The cleavage cocktail was drained and the resin washed with TFA. The peptide was precipitated in ice-cold Et_2_O. After centrifugation for 20 minutes at 4k rpm the supernatant was removed and the crude product was dissolved in water and ACN and purified via HPLC (10 % to 100 % ACN in 20 min). After lyophilisation the product was received as a beige solid (9.4 mg, 8.23 μmol, 3 %).

**MALDI-TOF** m/z: [M+H]^+^ 1141.4 (calc. 1141.63) (Isopeptide **1**) and [M+H]^+^ 1141.6 (calc. 1141.63) (Isopeptide **1_scr_**)


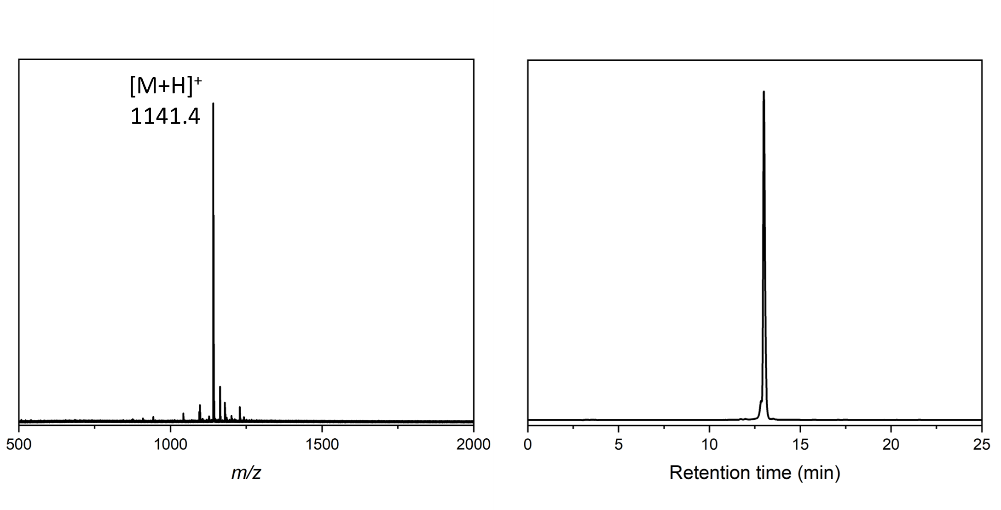


**Figure S5:** MALDI-TOF spectrum (left) and HPLC trace (right, 340 nm) of Isopeptide **1** with *t*_R_= 13.0 min.


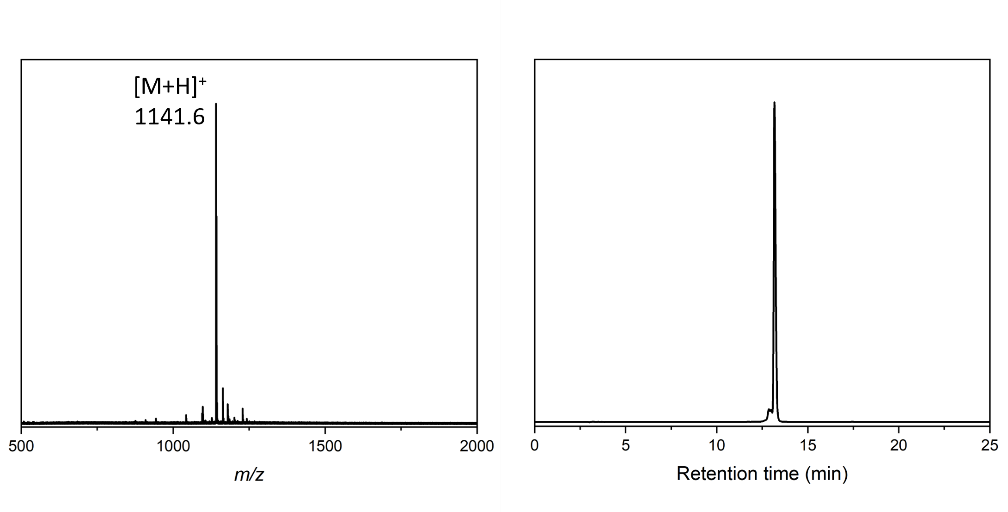


**Figure S6:** MALDI-TOF spectrum (left) and HPLC trace (right, 340 nm) of Isopeptide **1_scr_** with *t*_R_= 13.2 min.

**2.2 Synthesis of Linear Py-ISA 3**

The peptide was synthesized using a microwave assisted solid-phase peptide synthesizer. The Wang resin preloaded with Fmoc-Ala (0.705 g, 0.5 mmol) was covered and swollen in DMF for 1 h. Before every coupling step and as the final deprotection, the *N*‑terminal Fmoc group was cleaved by two deprotection steps using 20% piperidine in DMF (3 ml) for 15 s and 50 s at 75 °C and 90 °C, respectively. Fmoc-Ser(OH) and Fmoc-Ile (5 eq. in 1.25 ml) were coupled for 15 s and 110 s at 75 °C and 90 °C respectively using DIC (0.5 M) and Oxyma (1 M) in a total of 2.75 ml DMF. The resin was removed from the reaction vessel and washed with DMF and DCM.
1-Pyreneacetic acid (156.17 mg, 0.600 mmol, 1.2 eq.) and PyBOP (520.4 mg, 1.00 mmol, 2.0 eq.) were dissolved in dry DMF (5 mL) and DIPEA (0.35 mL, 2.00 mmol, 4 eq.) was added to the solution. To the ISA-loaded resin (0.50 mmol) dry DMF (3 mL) was added and the previously prepared 1-pyreneacetic acid-containing solution was added dropwise. The resin was shaken for 2.5 h at room temperature. The solution was drained and the resin was subsequently washed with DMF and DCM. The peptide sequence was cleaved from the resin by adding a mixture of TFA (4.75 mL, 95 %), TIPS (0.125 mL, 2.5%) and H_2_O (0.125 mL, 2.5 %) and shaken for 2.5 h at room temperature. The cleavage cocktail was drained and the resin washed with TFA. The peptide was precipitated in ice-cold Et_2_O. After centrifugation for 20 minutes at 4k rpm the supernatant was removed and the crude product was dissolved in water and ACN and purified via HPLC (10 % to 100 % ACN in 20 min). After lyophilisation the product was received as a colorless solid (26 mg, 48.9 μmol, 10 %).

**^1^H-NMR, COSY** (850 MHz, DMSO) δ/ppm = 8.41 (d, *J* = 9.15 Hz, 1H, H-9), 8.39 (d, *J* = 8.93 Hz, 1H, H-11), 8.28 (t, *J* = 7.28 Hz, 2H, H-29, H-5), 8.24 (d, *J* = 7.71 Hz, 1H, H-2), 8.20 (d, **J** = 9.17 Hz, 1H, H-8), 8.15 (s, 2H, H-34, H-3), 8.07 (d, *J* = 7.51 Hz, 1H, H-6), 8.05 (t, *J* = 8.50 Hz, 1H, H-17), 8.02 (d, *J* = 7.73 Hz, 1H, H-1), 7.95 (m, 1H, H-19), 4.37 (d, *J* = 15.00 Hz, 1H, H-10), 4.33 (q, *J* = 6.42 Hz, 1H, H-18), 4.27 (dd, *J* = 7.20 Hz, 8.95 Hz, 1H, H-12), 4.23 (d, *J* = 15.00 Hz, 1H, H-10’), 4.18 (m, 1H, H-21), 3.57 (m, 2H, H-19), 1.78 (m, 1H, H-13), 1.46 (m, 1H, H-14), 1.21 (d, *J* = 7.29 Hz, 3H, H-22), 1.12 (m, 1H, H-14’), 0.84 (d, *J* = 6.83 Hz, 3H, H-15), 0.78 (t, *J* = 7.43 Hz, 3H, H-16).

**ESI** m/z: [M+H]^+^ 532.2 (calc. 532.2), [2M+H]^+^ 1063.4 (calc. 1063.5).

**
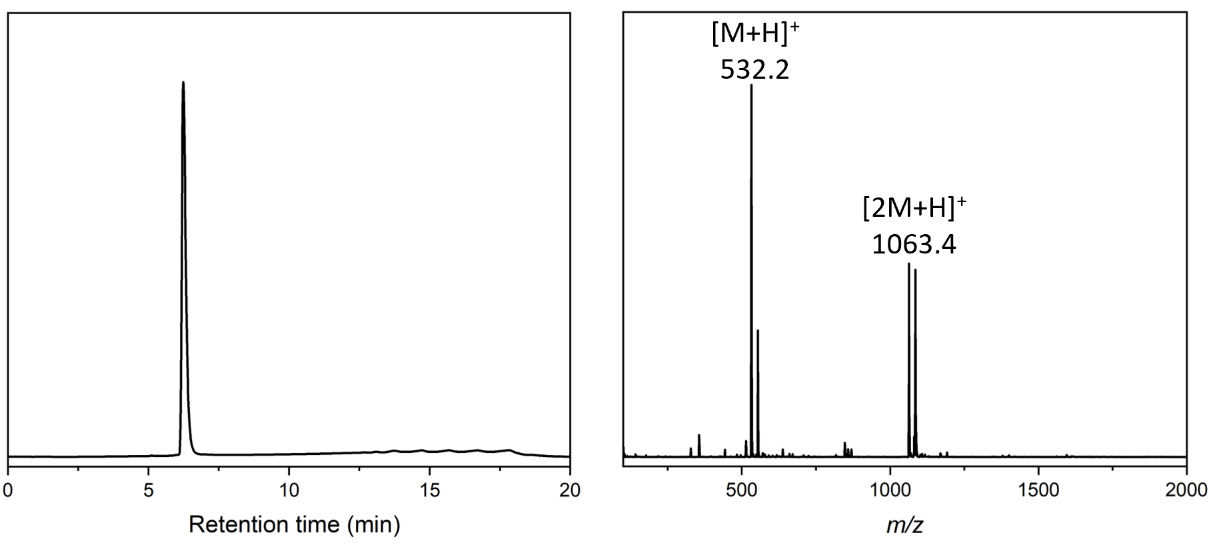
**

**Figure S7:** LC-MS data of linear Py-ISA **3**. **Left** LC trace (254 nm) with *t*_R_= 6.27. **Right** Convoluted ESI-MS spectrum.

**Figure S8:** ^1^H NMR spectrum (850 MHz, DMSO-*d*_6_, 298 K) of linear Py-ISA **3**.

**Figure S9:** ^1^H,^1^H COSY NMR spectrum (850 MHz, DMSO-d6, 298 K) of linear Py-ISA 3.

**3. Furin-Induced Transformation of Isopeptides**

**3.1. General procedure of HPLC Kinetic Analysis**

The furin-induced conversion of the isopeptide **1** and **1_scr_** was analyzed via an analytical HPLC kinetic study. The samples were prepared by adding the enzyme (1 nmol peptide/ U) to the isopeptide **1** (20 μM) in a buffered aqueous solution containing HEPES (100 mM), TCEP (1 mM) and CaCl_2_ (1 mM). The pH of the buffer was adjusted to 7.4 using 1M NaOH. The control sample was prepared by solving the isopeptide **1_scr_** in buffered aqueous solution without enzyme. Samples were measured after 0.5 h, 1h, 2h, 4h, 6h, 8h and 24h by mixing 50 μL of the sample with 50 μL of methanol. After centrifugation of 5 minutes at 12,000 rpm, 50 μL of the supernatant was injected in the analytical HPLC to monitor the conversion process.

_
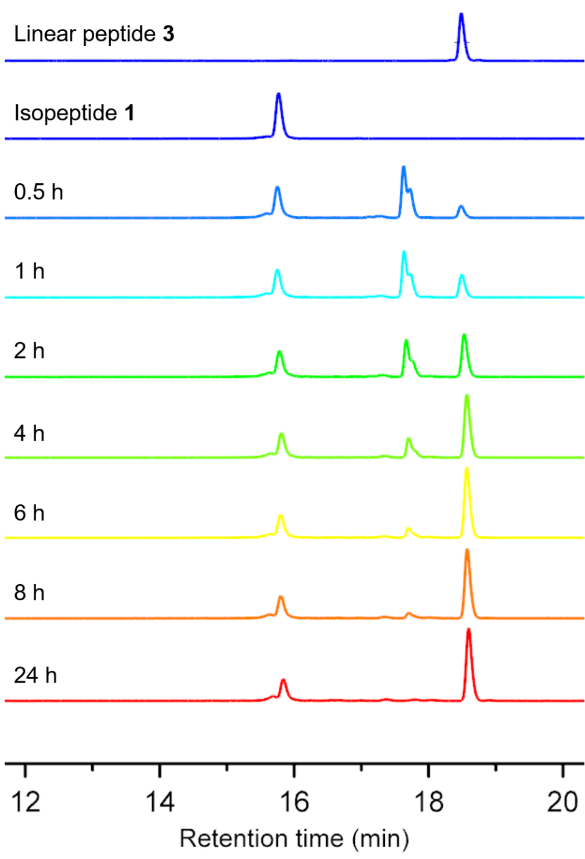
_

**Figure S10:** HPLC study of the kinetics of enzyme-induced conversion of isopeptide **1** (100 µM) in the presence of the protease furin (1 nmol peptide/ U) in buffered solution over time (HEPES (100 mM), CaCl_2_ (1 mM), TCEP (1 mM)).

**Scheme S2:** Mechanism of *O,N*-acyl rearrangement of free isopeptide **2** to linear peptide **3**.

**3.2. General Procedure of TEM Kinetic Analysis**

The furin-induced structure formation of isopeptide **1** was analyzed via TEM kinetic study. The samples were prepared by adding the enzyme (1 nmol/U) to the isopeptide **1** (20 μM) in a buffered aqueous solution containing HEPES (100 mM), TCEP (1 mM) and CaCl_2_ (1 mM). The pH of the buffer was adjusted to 7.4 using 1 M NaOH. TEM grids were prepared after 0.5 h, 1h, 2h, 4h, 6h, 8h and 24h by adding 3 μL by pipetting 3 μL of the solution onto a Formvar‑coated copper grid and incubating for 5 min. After the incubation, the solutions were removed with filter paper, and the grids were stained with 10 μL 4% uranyl acetate solution for 2.5 min. The grids were washed three times with MilliQ water and dried before being measured.


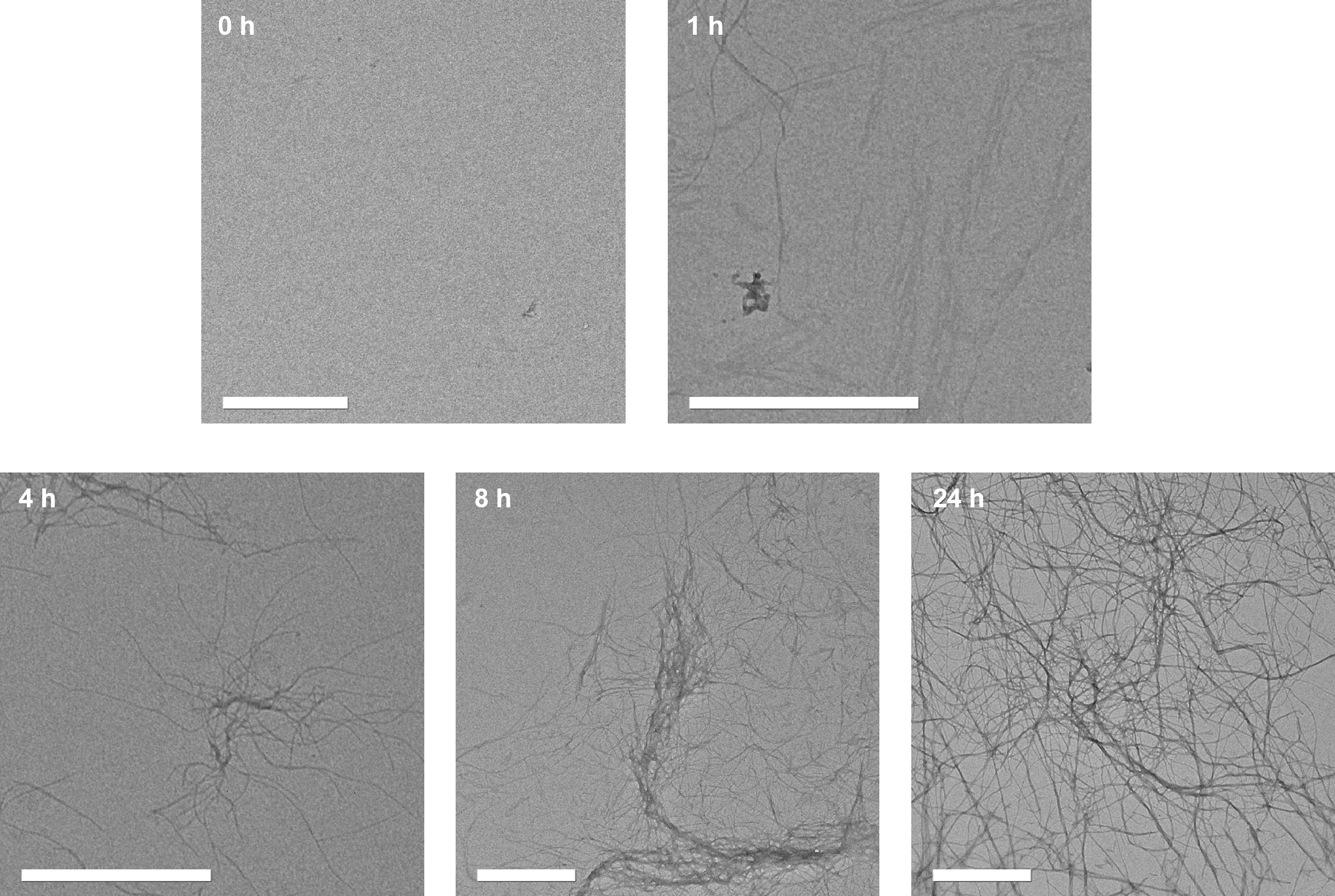


**Figure S11:** Dry-state TEM images of reaction solution of isopeptide **1** (200 µM) in the presence of furin in buffered solution (HEPES (100 mM), CaCl_2_ (1 mM), TCEP (1 mM)) after incubation for 0 h, 1 h, 4 h, 24 h at 37 °C. Scale bars 500 nm.

**4. Analysis of Secondary Structure and Self-Assembly Behavior**

**4.1. Temperature-dependent ^1^H-NMR Analysis of Linear Py-ISA 3**

For the temperature-dependent NMR analysis a solution of the linear peptide **3** was prepared containing 0.23 mg peptide in 0.5 ml deuterated solvent. The deuterated solvent consisted of a 9:1-mixture of phosphate buffer (50 mM) prepared from D_2_O and DMSO-d_6_. The sample was then immediately analyzed via ^1^H-NMR spectroscopy at 298 K and the temperature was increased in 5 °C steps to 353 K. At each temperature the sample was equilibrated for 20 min before the measurement.


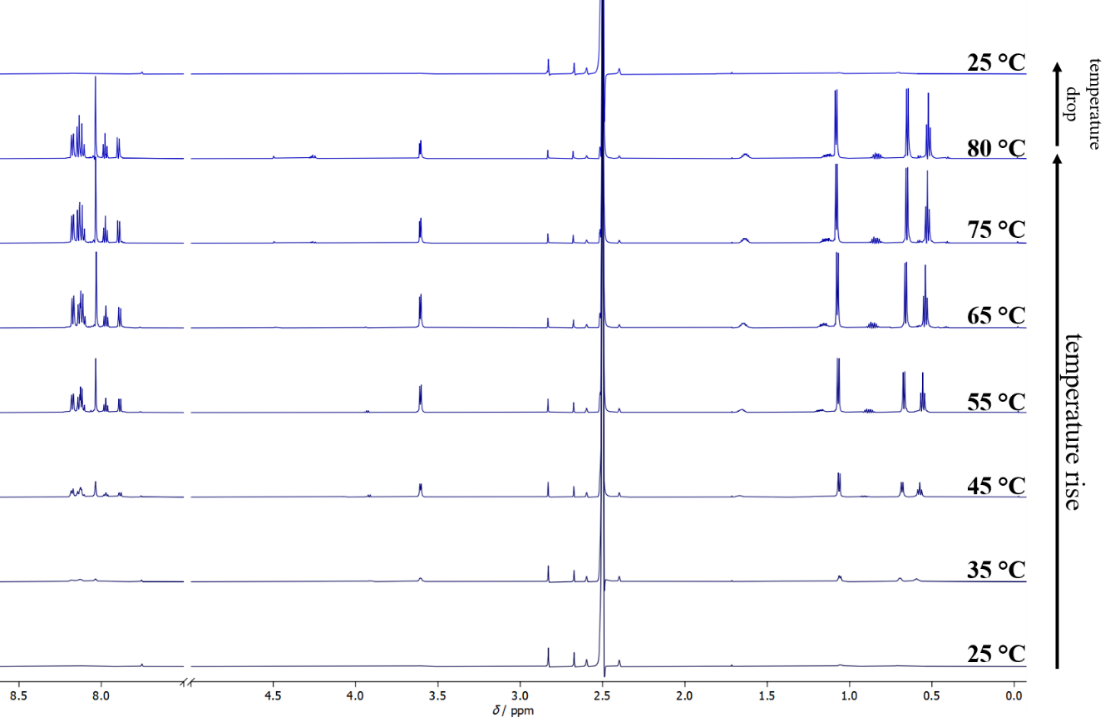


**Figure S12:** Temperature-dependent ^1^H-NMR analysis of linear Py-ISA **3** in phosphate buffer (50 mM) prepared from D_2_O and DMSO-*d*_6_ (9:1).

**4.2. CD Spectra of Py-ISA 3 and Kinked Assembly Precursor 1**

The linear peptide **3** or the kinked assembly precursor **1** were dissolved in phosphate buffer (10 mM, pH 7.4) to yield a peptide concentration of 100 µM. The solutions were sonicated to dissolve the peptides completely. The samples were subsequently measured, and the spectra were recorded at wavelengths from 380 to 180 nm with a bandwidth of 1 nm, data pitch of 0.2 nm, and scanning speed of 5 nm/min. The spectra were measured three times and accumulated.

**4.3. TEM Analysis of Linear Py-ISA 3 and Kinked Assembly Precursor 1**

The linear peptide **3** or the isopeptides **1** and **1_scr_** were dissolved in DMSO (1 mM) and diluted with DPBS at a ratio of 1:9 to yield a 100 μM peptide solution. The solution was incubated for 24 h and TEM grids were prepared by pipetting 3 μL of the solution onto a Formvar-coated copper grid and incubated for 5 min. After the incubation, the solutions were removed with filter paper, and the grids were stained with 10 μL 4% uranyl acetate solution for 2.5 min. The grids were washed three times with MilliQ water and dried before being measured.


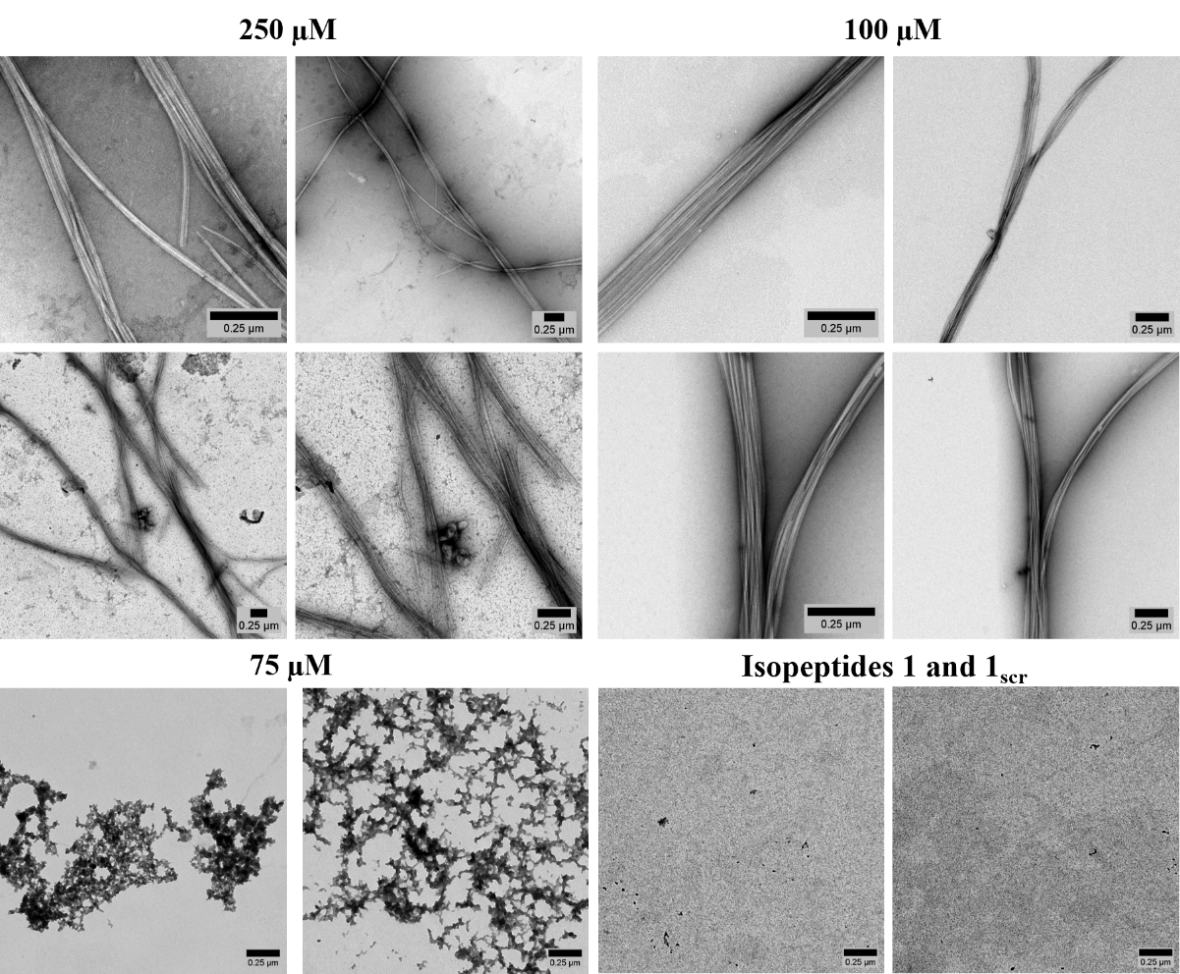


**Figure S13:** Dry-state TEM images showing nanofiber bundles and aggregates formed by linear peptide **3** (250 µM, 100 µM, 75 µM) in DPBS (pH 7.4) and DMSO (9:1) and dry-state TEM images of Isopeptides **1** and **1_scr_** showing the absence of aggregate or structure formation. Scale bar: 250 nm.

**4.4. Cryo-TEM Analysis of Linear peptide 3**

The linear peptide **3** was dissolved in DMSO (1 mM) and diluted with DPBS at a ratio of 1:9 to yield a 100 μM peptide solution. The solution was incubated for 24 h before 3 μL of the sample was used for imaging.

**4.5. Proteostat Aggregation Assay**

For the analysis of the critical aggregation concentration of the linear peptide **3** the commercial Proteostat protein aggregation assay kit by *Enzo Life Sciences, Inc.* was used. The peptide samples were prepared by diluting DMSO stock solutions of the linear peptide **3** in various concentrations (1 μM to 100 µM) and isopeptide **1** at a concentration of 250 µM with DPBS in a 1:9 ratio. The resulting peptide samples were incubated for 24 h at room temperature while shaking. The Proteostat working solution was prepared by mixing 0.52 μl of the Proteostat stock solution with 98.48 μl MilliQ water and 1 μl assay buffer. After the incubation, 27 μl of each peptide solution were mixed with 3 μl of the Proteostat working solution. 9 μl of each solution were pipetted into a well of a Greiner 384 flat black well plate (3 wells per sample of each peptide concentration). The plate was incubated in the dark for 15 min while shaking. The fluorescence intensity of the Proteostat dye was subsequently measured with an excitation and emission bandwidth of 20 nm and an emission wavelength of 600 nm after excitation at 550 nm.


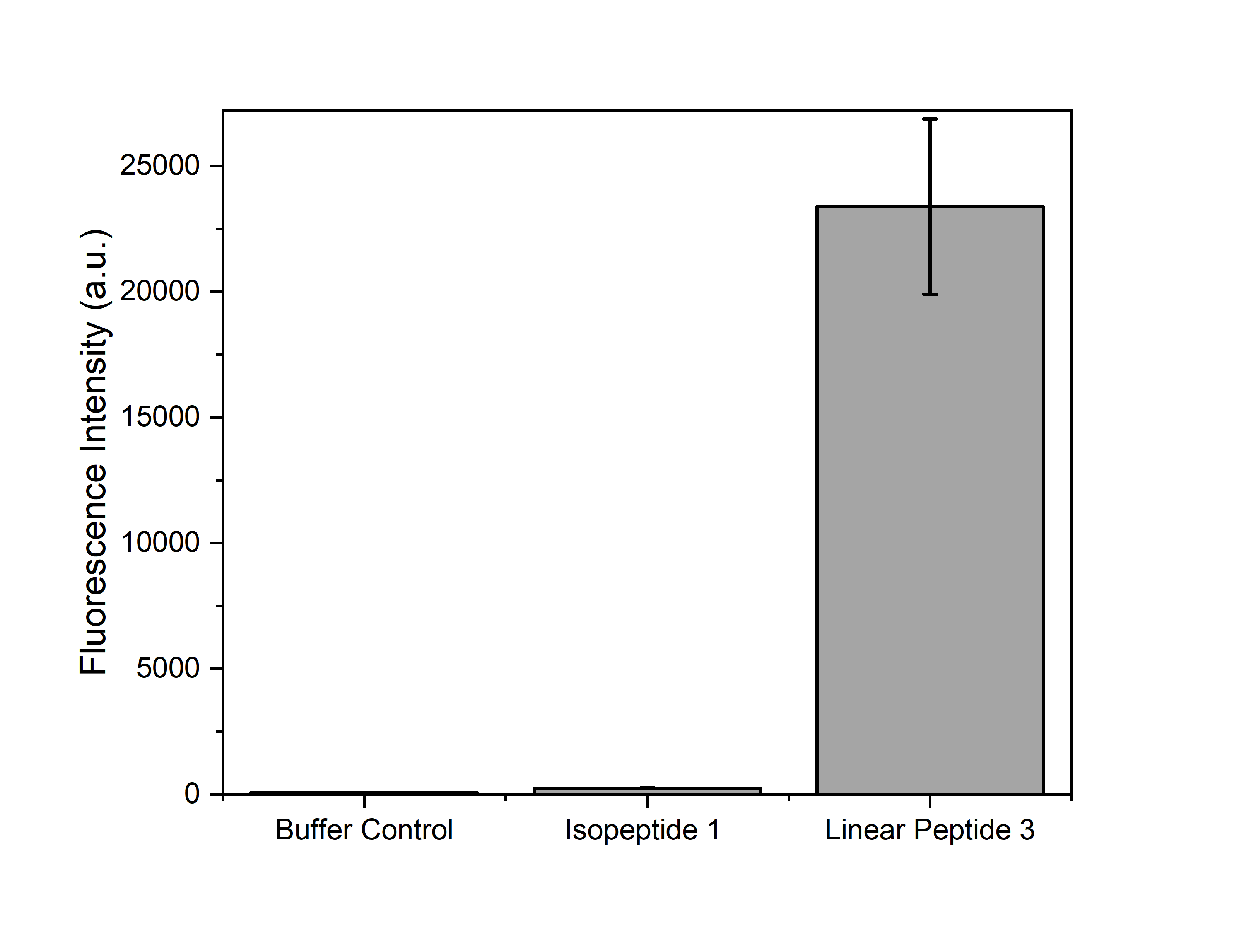


**Figure S14:** Proteostat aggregation assay of linear peptide **3**, isopeptide **1** in DPBS and DMSO (9:1) at a concentration of 250 μM as well as the DPBS buffer (10% DMSO) serving as control.

**4.6. AFM**

The linear peptide **3** was dissolved in DMSO (1 mM) and diluted with DPBS at a ratio of 1:9 to yield a 250 μM peptide solution. The solution was incubated for 24 the sample was used for imaging.


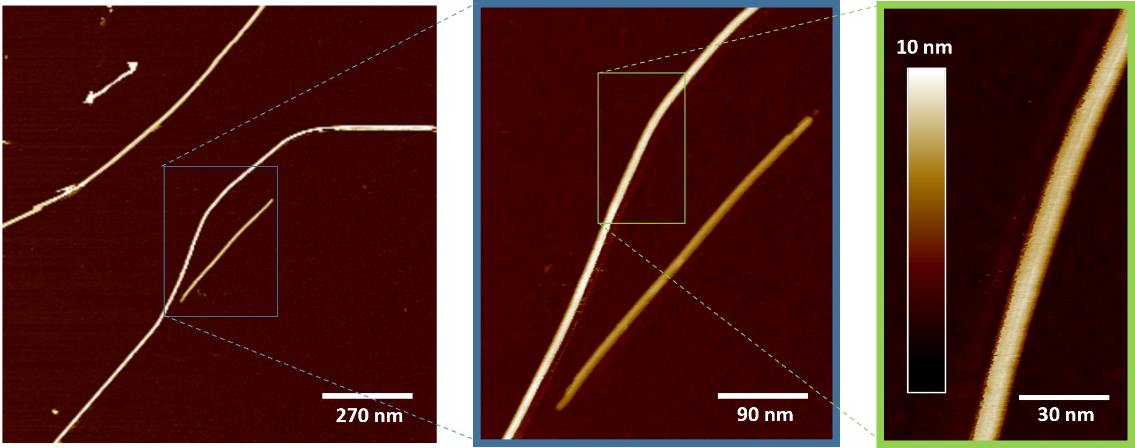


**Figure S15:** AFM images of peptide nanofibers formed by linear peptide **3** (250 µM) in DPBS (pH 7.4) and DMSO (9:1). Scale bar: 270 nm (left), 90 nm (middle) and 30 nm (right).
